# Characterization of Four Popular Sweet Cherry Cultivars Grown in Greece by Volatile Compound and Physicochemical Data Analysis and Sensory Evaluation

**DOI:** 10.3390/molecules20021922

**Published:** 2015-01-26

**Authors:** Maria V. Vavoura, Anastasia V. Badeka, Stavros Kontakos, Michael G. Kontominas

**Affiliations:** 1 Department of Chemistry, University of Ioannina, 45110-Ioannina, Greece; E-Mails: mvavoura@cc.uoi.gr (M.V.V.); abadeka@cc.uoi.gr (A.V.B.); 2 Department of Social Administration and Political Science, Democritus University of Thrace, 69100-Komotini, Greece; E-Mail: stkontakos@yahoo.gr; 3 Department of Chemistry, American University in Cairo, 11835-New Cairo, Egypt

**Keywords:** volatile compounds, physicochemical parameters, sensory attributes, cherry cultivars

## Abstract

Volatile compounds, physicochemical and sensory attributes of four sweet cherry cultivars (Canada giant, Ferrovia, Lapins and Skeena) grown in Northern Greece were determined. Eighteen volatile compounds were identified and semi-quantified in cherries using solid phase micro extraction in combination with gas chromatography/mass spectrometry (SPME-GC/MS). Carbonyl compounds were the most abundant in sweet cherry aroma, followed by alcohols, esters and hydrocarbons/terpenes. Cherry cultivars in order of increasing amounts of volatiles were: Lapins < Canada giant < Ferrovia < Skeena. Physicochemical parameters determined included: titratable acidity (TA), pH, total soluble solids (TSS), maturity index (MI) and total phenolic content (TPC). TA ranged between 0.21 and 0.28 g malic acid/100 g fresh weight (FW). The pH ranged between 3.81 and 3.96. TSS ranged between 13.00 and 16.00 °Brix. MI ranged between 51.8 and 75.0. TPC ranged between 95.14 and 170.35 mg gallic acid equivalents (GAE)/100 g FW. Sensory evaluation showed that cherry colour, in order of increasing intensity, was: Canada giant < Ferrovia < Lapins < Skeena. Respective order for cherry firmness was: Canada giant < Lapins ≤ Ferrovia < Skeena and for flavour: Lapins < Canada giant < Skeena ≤ Ferrovia. Correlation of volatiles to physicochemical and sensory attributes showed varying trends.

## 1. Introduction

Sweet cherries of the genus *Prunus avium* (family *Rosaceae*) are non-climacteric stone fruit, mainly grown in temperate climate countries. They are an important crop in Greece, with an annual production of 45,000–60,000 tons (7%–8% of EU production). Greece ranks 12 in world cherry production [1]. Even though sweet cherry trees are cultivated all over Greece (the mainland and the islands), 65%–70% of the cherry production concentrates in northern Greece in the greater area of Imathia and Pella [1].

A ripe cherry fruit has bright shiny pale to deep red or even purple colour, usually with a thin skin. Due to their colour, aroma, taste and health beneficial antioxidant properties, cherries are greatly appreciated worldwide [2,3]. Among the classical sweet cherry cultivars grown in Greece, the Ferrovia cultivar gives a large, red, shiny skin, heart-shaped fruit of firm texture destined for fresh consumption. The cultivar originates from the area of Bari (Puglia, Italy) but has adapted very well in various geographical areas of Greece. Fruit ripens in early June. The fruit flesh is pink in colour, juicy, with a strong adherence to the stone. It has a very pleasant flavour of intermediate sweetness [4]. The Lapins cultivar also gives a very large, red, shiny skin, heart-shaped fruit of firm texture destined for fresh consumption. This cultivar was developed by K.O. Lapin at the Agricultural Research Station in Summerland, British Columbia in Canada. The fruit ripens in late May to early June. The fruit flesh is very juicy of dark red colour.

Among the newer promising cultivars grown in Greece, the Skeena cherry cultivar gives a large, red, shiny skin, kidney-shaped fruit of very firm texture destined for fresh consumption. Finally, the Canada giant cultivar gives a large, red, shiny skin, heart-shaped fruit of firm texture to be consumed fresh. Both the Skeena and Canada giant cultivars were also developed in Summerland, Canada. Greece has the comparative advantage of early sweet cherry ripening by 10–15 days as compared to the rest of Europe, with the exception of Turkey.

In terms of sensory quality, the main cherry attributes include skin colour, sweetness (sugar content), sourness (organic acid content), fruit firmness, fruit weight, and aroma even though the compounds contributing to fruit odour comprise a very small portion (0.01%–0.001%) of the fruit fresh weight (FW) [5]. Aroma is one of the most valued attributes of sweet cherries which may influence consumer acceptance of the fruit. On the other hand, the total soluble solids (TSS)/titratable acidity (TA) ratio, known as maturity index, directly affects the perception of sweetness and flavour and thus, consumer acceptance of the fruit [2]. Cherries are also a good source of phenolic compounds (*ca.* 150 mg GAE/kg FW) made up mainly of hydroxycinnamates, anthocyanins, flavan-3-ols and flavonols [6]. Such compounds are responsible for the strong antioxidant activity of cherry fruit [7].

It has been reported that TSS and TA are closely related to the intensity of cherry flavour and consumer acceptability increases with high TSS and TA levels [2,8]. TA has also been reported to be dependent on cherry cultivar with levels of 0.4%–1.5%.

It is well accepted that the aroma of fruit is the combined result of a complex mixture of esters, alcohols, aldehydes, organic acids, ketones, terpenoid compounds, *etc.* [9]. Methods used to analyze such complex mixtures include static and dynamic head space analysis, supercritical CO_2_ extraction, and solid phase micro extraction (SPME) in combination with gas chromatography/mass spectrometry (GC/MS) [3,10,11]. Among these, SPME has proven to be a simple, rapid, solvent-free and effective method to analyze fruit volatiles [5,12].

In addition to the determination of volatiles, sugar and organic acid content, it is appropriate that these attributes be correlated to sensory evaluation attributes (taste, odour, colour and texture) to characterize the overall quality of sweet cherry fruit [13]. 

Based on the above, the objective of the present work was to determine the volatile composition and physicochemical attributes of four popular cherry cultivars grown in Greece and to correlate these to respective sensory evaluation data.

## 2. Results and Discussion

### 2.1. Physicochemical Parameter Data

Results for titratable acidity (TA), pH, total soluble solids (TSS), maturity index (MI) and total phenolic content (TPC) are given in Table 1.

**Table 1:** Physicochemical parameter data of four sweet cherry cultivars.

Cultivar	TA (g malic acid/100 g FW)	pH	TSS (°Brix)	MI	TPC (mg GAE/100 g FW)
**Canada giant**	0.28 ± 0.01 ^b^	3.91 ± 0.00 ^b^	14.50 ± 0.05 ^b^	51.8 ^a^	95.14 ± 3.64 ^a^
**Lapins**	0.20 ± 0.00 ^a^	3.96 ± 0.01 ^c^	13.00 ± 0.03 ^a^	65.0 ^c^	135.86 ± 13.94 ^b^
**Ferrovia**	0.27 ± 0.00 ^b^	3.81 ± 0.00 ^a^	16.00 ± 0.06 ^d^	59.3 ^b^	124.63 ± 0.62 ^b^
**Skeena**	0.21 ± 0.01 ^a^	3.91 ± 0.00 ^b^	15.75 ± 0.01 ^c^	75.0 ^d^	170.35 ± 3.52 ^c^

^a–d^ different lower case letters in a given column indicate significant differences (*p* < 0.05) as observed by Duncan’s multiple comparison tests.

TA ranged between 0.21 for the Skeena cultivar to 0.28 g malic acid/100 g FW for the Canada giant cultivar. Significant differences (*p* < 0.05) were observed between both Lapins/Skeena and Ferrovia/Canada giant cultivars. Differences in TA between cherry cultivars have been previously reported among Picota type and Sweetheart cultivars [3] and between Ambrunes and Pico Colorado cultivars [10].

The pH ranged from 3.81 for the Ferrovia cultivar to 3.96 for the Lapins cultivar. Significant differences (*p* < 0.05) in pH were observed between both Canada giant/Skeena and Lapins, Ferrovia cultivars. Serradilla *et al.* [3] reported cherry pH values between 3.76 and 4.36 while Bernalte *et al.* [10] reported pH values between 4.15 and 4.29 for above mentioned sweet cherry cultivars. Likewise, Souza *et al.* [14] and Wen *et al.* [15] reported pH values between 4.07 and 4.09 and between 4.04 and 4.08 for Brazilian and Chinese sweet cherries, respectively.

TSS ranged between 13.00 °Brix for the Lapins cultivar to 16.00 °Brix for the Ferrovia cultivar. Significant differences (*p* < 0.05) in TSS were observed among all cultivars studied. Serradilla *et al.* [3] reported TSS values between 15 and 20 °Brix for three Picota type and Sweetheart cultivars while Bernalte *et al.* [10] reported even higher TSS values between 19.5 and 21.4 °Brix for Ambrunes and Pico Colorado cherry cultivars. Likewise Souza *et al.* [14] and Wen *et al.* [15] reported TSS values 18.09 to 19.27 and 17.77 to 19.77 °Brix for Brazilian and Chinese sweet cherries, respectively. Crisosto *et al.* [2] suggested a TSS value above 14 °Brix for cherries to be acceptable for marketing. In the present study the Lapins cultivar had a TSS of 13 °Brix, below the above suggested limit. TSS values were also in general agreement with those of Souza *et al.* [3], Girard and Kopp [16] and Gonzalez-Gomez *et al.* [17].

The maturity index (MI = TSS/TA) ranged from 51.8 for the Canada giant cultivar to 75.0 for the Skeena cultivar. Significant differences (*p* < 0.05) were observed among all four cultivars studied. These values are substantially higher than those of Serradilla *et al.* [3], Bernalte *et al.* [10] and Souza *et al.* [14]. Such differences in MI may be related to differences in maturity during fruit collection.

The total phenolic content ranged from 95.14 (for the Canada giant cultivar) to 170.35 mg GAE/100 g FW (for the Skeena cultivar). It is noteworthy to mention that the latter cultivar is of specific interest due to its very high phenolic content rendering it a fruit of great antioxidant activity expected to exert a beneficial health effect upon consumption [18]. Differences in TPC between the Ferrovia and Lapins cultivars were non-significant (*p* > 0.05).

Serradilla *et al.* [3] reported TPC values for Picota type and Sweetheart cherry cultivars ranging from approximately 60 to 154 mg GAE/100 g FW. It has also been reported that phenolic compounds contribute to fruit quality by modifying colour, aroma, flavour as well as sensory properties such as bitterness and astringency [19,20].

### 2.2. Volatile Compounds

Volatiles identified and semi-quantified in the four cherry cultivars using SPME are given in Table 2. A total of 18 compounds belonging to the groups: alcohols, aldehydes, ketones, hydrocarbons/terpenes and esters were identified and semi-quantified using 4-methyl-2-pentanone as the internal standard. Almost all compounds identified have been reported previously in fresh sweet cherry fruit [3,5,10,16,21]. 

Carbonyl compounds showed the most abundant signals present in sweet cherry aroma corresponding to amounts ranging from 14.75 µg/kg in the Lapins cultivar to 34.62 µg/kg in the Ferrovia cultivar. Carbonyl compounds identified were linear and aromatic. The most abundant carbonyl compound was 2-propanone followed by 2-hexenal and acetaldehyde. 2-Propanone ranged from 5.15 µg/kg for the Lapins cultivar to 15.12 µg/kg for the Ferrovia cultivar. 2-Hexenal ranged from 2.16 µg/kg for the Canada giant cultivar to 10.39 µg/kg for the Skeena cultivar. Finally, acetaldehyde ranged from 2.27 µg/kg for the Skeena cultivar to 5.55 µg/kg for the Ferrovia cultivar. 

Carbonyl compounds are known to be among the most important aroma compounds of sweet cherry fruit [5,10,16,21]. 2-hexenal and hexanal are known as “green leaf volatiles” and have a very low perception threshold [22]. In contrast to the results of Serradilla *et al.* [3] 2-propanone was the most abundant volatile compound identified in all four cherry cultivars.

**Table 2:** Identification and semi—quantification of volatile compounds (data are expressed as µg of I.S./kg fruit).

Compound	RI_EXP_ *	RI_NIST_ **	Canada Giant	Ferrovia	Lapins	Skeena
**Carbonyl Compounds**			21.48	34.62	14.75	26.47
Acetaldehyde	<500	408	3.96^B^ ± 1.68 ***	5.55^D^ ± 0.54	5.27^C^ ± 0.21	2.27^A^ ± 0.34
2-Propanone	<500	500	14.15^C^ ± 6.45	15.12^D^ ± 2.22	5.15^A^ ± 1.94	8.18^B^ ± 3.68
Hexanal	801	798	0.71^B^ ± 0.43	2.72^D^ ± 0.19	0.62^A^ ± 0.31	2.18^C^ ± 1.16
2-Hexenal	856	859	2.16^A^ ± 1.42	10.36^C^ ± 0.18	2.60^B^ ± 1.62	10.39^C^ ± 6.31
2,4-Hexadienal	916	916	n.d.	0.29^A^ ± 0.03	n.d.	0.43^B^ ± 0.25
Benzaldehyde	974	973	0.45^A^ ± 0.07	0.50^B^ ± 0.13	1.06^C^ ± 0.34	2.95^D^ ± 0.41
Nonanal	1108	1103	0.05^A^ ± 0.00	0.08^B^ ± 0.01	0.05^A^ ± 0.03	0.07^B^ ± 0.01
**Alcohols**			9.97	5.56	7.72	22.21
Ethanol	<500	427	7.99^D^ ± 1.29	3.59^A^ ± 0.61	6.66^B^ ± 0.39	6.98^C^ ± 1.04
2-Butanol	600	608	0.08^A^ ± 0.05	0.09^B^ ± 0.06	n.d.	n.d.
3-Methyl-3-buten-1-ol	731	724	0.12^B^ ± 0.01	0.16^C^ ± 0.01	0.13^B^ ± 0.00	0.85^A^ ± 0.23
3-Methyl-butan-1-ol	733	743	0.11^A^ ± 0.02	n.d.	n.d.	0.32^B^ ± 0.09
3-Methyl-2-buten-1-ol	772	770	0.13^A^ ± 0.02	0.20^C^ ± 0.01	0.15^B^ ± 0.02	0.96^D^ ± 0.26
2-Hexen-1-ol	864	865	1.02^B^ ± 0.20	1.04^B^ ± 0.64	0.78^A^ ± 0.55	7.57^C^ ± 1.48
1-Hexanol	867	870	0.53^A^ ± 0.37	0.49^A^ ± 0.34	n.d.	2.25^B^ ± 0.37
Benzyl Alcohol	1045	1045	n.d.	n.d.	n.d.	3.29 ± 1.15
**Other Compounds**						
2-Hexenyl acetate	1012	1014	0.08^A^ ± 0.07	n.d.	n.d.	0.13^B^ ± 0.08
2-Methyl-1,3-butadiene	508	520	n.d.	n.d.	0.28^A^ ± 0.09	0.66^B^ ± 0.17
d-Limonene	1044	1039	0.03^A^ ± 0.01	0.06^B^ ± 0.01	0.04^A^ ± 0.01	n.d.

I.S. = internal standard; * RI_EXP_ = Retention Index experimentally determined data; ** RI_NIST_ = Retention Index literature data NIST 05; *** Mean of tree repetitions is reported; n.d. = not detected; ^A–D^ different capital letters within a given row indicate significant differences (*p* < 0.05) as observed by Duncan’s multiple comparison tests.

Alcohols gave the second most abundant signals, corresponding to amounts ranging from 5.56 for the Ferrovia cultivar to 22.21 µg/kg for the Skeena cultivar. Alcohols identified were linear, branched and aromatic. The most abundant alcohol was ethanol: 7.99 µg/kg in the Canada giant cultivar, 6.98 µg/kg in the Skeena cultivar, 6.66 µg/kg in the Lapins cultivar and 3.59 µg/kg in the Ferrovia cultivar. In a similar study, Serradilla *et al.* [3] identified (*E*)-2-hexen-1-ol as the main alcohol present in Picato type and Sweetheart sweet cherries in Spain. 2-Hexen-1-ol was also identified in the present study in rather large amounts (7.57 µg/kg) only in cherries of the Skeena cultivar. 2-Hexen-1-ol is related to green notes and fresh green odours associated with fruit and vegetables [23]. Along with hexanal and 2-hexenal they predominate flavour volatiles in sweet cherries [16]. Of the aromatic alcohols, benzyl alcohol was found only in the Skeena cherry cultivar. This compound may thus, be used as a marker for the differentiation of the Skeena cultivar from the rest three cultivars.

Esters were found in very small concentrations in all cherry cultivars. Finally, other minor compounds such as alkenes (2-methyl-1,3-butadiene) and terpenes (D-limonene) were found in very small amounts in individual cherry cultivars.

The most representative compounds in the Skeena cultivar were C_6_ and aromatic compounds. Total alcohols or specifically 2-hexen-1-ol, 1-hexanol, benzyl alcohol and benzaldehyde may differentiate the Skeena cultivar. Likewise, total carbonyls may differentiate the Lapins cultivar from the rest of the cultivars. Furthermore, hexanal and 2-hexenal may differentiate Ferrovia and Skeena from the other 2 cultivars.

Girard and Kopp [16] studied the volatiles of 12 sweet cherry cultivars using dynamic headspace GC and reported that of the 50 compounds identified, (*E*)-2-hexenol, benzaldehyde, hexanal and (*E*)-2-hexanal were predominant flavour volatiles which could be used to differentiate commercial and new cherry selections into various subgroups. Likewise, Sun *et al.* [24] studied five sweet cherry cultivars grown in China and reported that the most represented classes of compounds in all five cultivars were C_6_ compounds and aromatic compounds. Hexanal, (***E***)-2-hexanal, 1-hexanol, (*E*)-2-hexen-1-ol, benzaldehyde and benzyl alcohol were the predominant volatiles in all five cultivars studied. C_6_ compounds are known to have a characteristic “green leaf” odour. Zhang *et al.* [5] identified 27 volatiles in sweet cherries in China and reported that hexanal, (*E*)-2-hexanal, benzaldehyde, (*E*)-2-hexen-1-ol, ethyl acetate and hexanoic acid ethyl ester were the characteristic aroma components of sweet cherry fruit.

According to Reineccius [25] fruit (cherry) flavour is not present during early fruit formation but develops entirely during a brief ripening period. During this period metabolism of the fruit changes to catabolism, and flavour formation begins. Minute amounts of carbohydrates, lipids and amino acids are enzymatically converted to simple sugars or acids and volatile compounds. 

Volatiles are produced either as the direct products of a particular metabolic pathway, or as a result of interactions between pathways or end products. A large number of volatile compounds are produced which contribute to the flavour (aroma) of a ripe fruit.

#### 2.2.1. Volatiles Derived from Lipids

Volatile flavour compounds may be formed from lipids via several different pathways. The primary pathways include mainly β-oxidation, and oxidation via lipoxygenase enzymes. Linolenic acid is metabolized via the lipoxygenase catalyzed oxidation producing 2-(*E*)- or 3-(*E* or *Z*)-hexenal which in turn are reduced to 2-(*E*)- or 3-(*E* or *Z*)-hexenol, responsible for the formation of respective hexenyl esters.

Linoleic acid, via the same pathway produces hexanal which is reduced to hexanol, responsible for the formation of hexyl esters. Through β-oxidation, linoleic acid produces hexanoic acid which may be converted to butanoic or pentanoic acid, responsible for the formation of the respective esters (hexanoate, pentanoate, butanoate, butyl and pentyl). The widest variety of flavour compounds formed from lipids arises via lipoxygenase activity. Many of the aliphatic esters, alcohols, acids, and carbonyls found in fruit are derived from the oxidative degradation of linoleic and linolenic acids. Galliard and Matthew [26] reported that only the 9- and 13-hydroperoxides are formed (95:5 ratio, respectively) via enzymic action. Later on, Galliard *et al.* [27] showed that only the 13-hydroperoxide makes the greater contribution to flavour. Acids and ketones as well as other intermediates in the oxidation process are readily converted to alcohols, aldehydes, and esters by other enzyme systems in the plant. Both α- and β-oxidation pathways have been demonstrated to exist thereby providing a wide range of volatiles for further conversion to flavour compounds [28].

Given the above, one may question the importance of these pathways since there is very little lipid in fruits. To address this question, one must recognize that many aroma compounds have low sensory thresholds (ppm). It takes only a small amount of precursor to yield ppm quantities of aroma compounds. Second, there is a significant quantity of linoleic and linolenic acid in plant chloroplasts. As fruits ripen, they lose their green colour due to chloroplast degradation, which then releases membrane lipids which are rich in these key aroma precursors. These precursors then enter the pathways described above to form a large variety of esters and carbonyls that characterize the aroma of many fruits. Based on ANOVA analysis it is shown that volatile compounds that come from fatty acid oxidation promoted by lipoxygenase are significantly more abundant in the Skeena cherry cultivar followed by the Ferrovia cultivar.

#### 2.2.2. Volatiles Derived from Aminoacids 

Aminoacid metabolism generates aromatic, aliphatic, and branched chain alcohols, acids, carbonyls, and esters that are important to the flavour of fruit. 1-Hexanol, 3-methylbutan-1-ol and 3-methyl-2-buten-1-ol are products of the deamination of the relevant α-aminoacids through oxidation reactions that occur during aerobic storage of cherry fruit [29]. Yu *et al.* [30,31,32] demonstrated that valine, leucine, alanine and aspartic acid can be converted to short chain carbonyls by tomato extracts. Radioactive labeling studies have shown that valine and leucine are transformed into branched chain flavour compounds that are essential to banana flavour (2-methyl propyl esters and 3-methyl butyl esters, respectively). The initial step in such transformations is deamination of the aminoacid followed by decarboxylation. Various reductions and esterifications then lead to a number of volatiles that are significant to fruit flavour (acids, alcohols, and esters). Further work has shown that aminoacids play a role in apple flavour as well. For example, isoleucine is the precursor of 2-methylbutyl and 2-methylbutenyl esters in apples [33,34]. The benzoic acids may be further transformed by esterification to yield benzyl esters and by reduction to yield various benzaldehydes and benzyl alcohols. Decarboxylation would yield phenols.

#### 2.2.3. Volatiles Derived from Carbohydrates

A large variety of volatile flavours can also be produced through carbohydrate metabolism. It has been well established that plants obtain all of their energy directly from photosynthesis. The photosynthetic pathways involve conversion of CO_2_ into sugars that are then metabolized into lipids and amino acids. Therefore, one may state that nearly all plant flavours come indirectly from carbohydrate metabolism. However, there are certain flavour constituents that come directly from carbohydrate metabolism. Terpenes, for example, derive both from carbohydrate and lipid metabolism. Terpenes are classified as monoterpenes containing two isoprene units (10 carbons), sesquiterpenes containing three isoprene units (15 carbons) and diterpenes containing four isoprene units (20 carbons). Of these groups, the monoterpenes, and more specifically, the oxygenated monoterpenes, are considered most important to the aroma of certain fruits, e.g., citrus products. Limonene, a monoterpene hydrocarbon possessing little odour, is the major terpene in most citrus oils accounting for up to 95% of some oils. The oxygenated terpenes, often comprising less than 5% of the oil, generally provide the characteristic flavour of different citrus species. For example, citral is considered the flavour impact component of lemon oil [25]. All four cherry cultivars were rich in ethanol which most probably derives from glycolysis [21].

It should be noted that besides volatile compounds, sweet cherry aroma is also dependent on non-volatiles *i.e.*, alcohols, terpenoids, norisoterpenoids, organic acids, *etc.*, bound in the form of glycosides [15]. These authors documented, however, that the latter are of the same nature as the first. They reported that regarding prediction of aroma, the free volatiles showed a fresh-green, citrus and floral aroma, whereas the bound volatiles were odourless.

### 2.3. Sensory Evaluation

Sensory evaluation data are shown in Table 3.

**Table 3:** Sensory evaluation data for the four cherry cultivars.

	Canada Giant	Skeena	Lapins	Ferrovia
flavour	3.75 ± 0.05^B^	3.90 ± 0.05^C^	3.63 ± 0.04^A^	3.98 ± 0.06^C^
texture	3.38 ± 0.05^A^	4.38 ± 0.06^C^	3.88 ± 0.03^B^	3.95 ± 0.05^B^
colour	4.03 ± 0.06^A^	4.70 ± 0.04^D^	4.50 ± 0.09^C^	4.17 ± 0.04^B^

^A–D^ different capital letters within a given row indicate significant differences (*p* < 0.05) as observed by Duncan’s multiple comparison tests.

With regard to appearance and colour, the Canada giant cherries were the largest (*ca.* 2.9 cm in diameter) as compared to all other cultivars. They were the lightest in red colour with a light coloured flesh. Regarding texture they were the least firm and regarding flavour they were sweet and slightly sour. The Skeena cultivar cherries were deep red in colour, both in skin and flesh (*ca.* 2.5 cm in diameter), the most firm in texture, sweet and slightly sour in flavour. The Lapins cultivar cherries (*ca.* 2.5 cm in diameter) were lighter (*p* < 0.05) in red colour (skin and flesh) than those of the Skeena cultivar. They were quite firm in texture and juicier than all others. The Ferrovia cultivar cherries were slightly darker (*p* < 0.05) in colour as compared to those of the Canada giant cultivar. Berries were non-homogeneous in colour (*ca.* 2.6 cm in diameter). Their flesh was quite light in colour. They were firm in texture, sweet and slightly sour in flavour.

In order of decreasing red colour cherry cultivars were: Skeena > Lapins > Ferrovia > Canada giant. Respective order for firmness was: Skeena > Ferrovia ≥ Lapins > Canada giant and for flavour was: Ferrovia ≥ Skeena > Canada giant > Lapins.

Other relevant studies also found significant differences between cherry cultivars studied as well as cherry ripening stages [3,10,35]. Ross *et al.* [36] evaluated firmness of “Selah” and “Skeena” cultivar sweet cherries using sensory difference testing (paired comparison test) and reported significant differences between the two for cherries of different firmness categories.

According to Chauvinn *et al.* [35] for cherry quality determination, the most appropriate method depends on the specific attribute of interest. Sensory evaluation techniques are the most effective for flavour/taste and colour attributes while analytical measurements are more effective for texture/firmness determination.

### 2.4. Correlation between Volatile Compounds and Physicochemical/Sensory Attributes

Linear correlation of volatile compounds of the four cherry cultivars with physicochemical and sensory attributes is presented in Table 4, Table 5, Table 6 and Table 7.

**Table 4:** Correlation between volatile compounds and physicochemical/sensory attributes in the Canada giant cultivar.

CANADA GIANT	Flavour	Texture	TA	pH	TSS	TPC	Colour
Acetaldehyde	−0.327	−0.655	- *	0.866	- *	0.500	−0.655
Ethanol	−0.327	−0.655	- *	0.866	- *	0.500	−0.655
2-Propanone	−0.327	−0.655	- *	0.866	- *	0.500	−0.655
2-Butanol	−0.327	−0.655	- *	0.866	- *	0.500	−0.655
3-Methyl-3-buten-1-ol	−0.327	−0.655	- *	0.866	- *	0.500	−0.655
3-Methylbutan-1-ol	−0.344	−0.668	- *	0.875	- *	0.515	−0.668
3 Methyl-2-buten-1-ol	−0.315	−0.645	- *	0.860	- *	0.489	−0.645
Hexanal	−0.327	−0.655	- *	0.866	- *	0.500	−0.655
2-Hexenal	−0.327	−0.655	- *	0.866	- *	0.500	−0.655
2-Hexen-1-ol	−0.326	−0.654	- *	0.865	- *	0.499	−0.654
1-Hexanol	−0.327	−0.655	- *	0.866	- *	0.500	−0.655
Benzaldehyde	−0.324	−0.652	- *	0.864	- *	0.497	−0.652
2-Hexenyl acetate	−0.323	−0.651	- *	0.864	- *	0.496	−0.651
D-Limonene	−0.327	−0.655	- *	0.866	- *	0.500	−0.655
Nonanal	−0.217	−0.564	−0.993	0.803	0.993	0.397	−0.564

* The value of the correlation coefficient was found to be equal to one which is statistically unrealistic, indicative however, of a very strong correlation.

**Table 5:** Correlation between volatile compounds and physicochemical/sensory attributes in the Ferrovia cultivar.

FERROVIA	Flavour	Texture	TA	pH	TSS	TPC	Colour
Acetaldehyde	- *	- *	- *	−0.500	- *	−0.500	−0.596
Ethanol	- *	- *	- *	−0.500	- *	−0.500	−0.596
2-Propanone	- *	- *	- *	−0.500	- *	−0.500	−0.596
2-Butanol	- *	- *	- *	−0.504	- *	−0.496	−0.600
3-Methyl-3-buten-1-ol	- *	- *	- *	−0.500	- *	−0.500	−0.596
3-Methyl-2-buten-1-ol	- *	- *	- *	−0.466	- *	−0.533	−0.565
Hexanal	- *	- *	- *	−0.500	- *	−0.500	−0.596
2-Hexenal	- *	- *	- *	−0.500	- *	−0.500	−0.596
2-Hexen-1-ol	- *	- *	- *	−0.500	- *	−0.500	−0.596
1-Hexanol	- *	- *	- *	−0.499	- *	−0.501	−0.595
2,4-Hexadienal	- *	- *	- *	−0.507	- *	−0.493	−0.603
Benzaldehyde	- *	- *	- *	−0.500	- *	−0.500	−0.596
D-limonene	- *	- *	- *	−0.500	- *	−0.500	−0.596
Nonanal	- *	- *	- *	−0.518	- *	−0.481	−0.613

* The value of the correlation coefficient was found to be equal to one which is statistically unrealistic, indicative however, of a very strong correlation.

**Table 6:** Correlation between volatile compounds and physicochemical/sensory attributes in the Lapins cultivar.

LAPINS	Flavour	Texture	TA	pH	TSS	TPC	Colour
Acetaldehyde	0.722	0.722	- *	0.501	0.501	−0.501	- *
Ethanol	0.721	0.721	- *	0.500	0.500	−0.500	- *
2-Propanone	0.721	0.721	- *	0.500	0.500	−0.500	- *
2-Methyl-1,3-butadiene	0.721	0.721	- *	0.500	0.500	−0.500	- *
3-Methyl-3-buten-1-ol	0.721	0.721	- *	0.500	0.500	−0.500	- *
3 Methyl-2-buten-1-ol	0.731	0.731	- *	0.513	0.513	−0.513	- *
Hexenal	0.720	0.720	- *	0.499	0.499	−0.499	- *
2-Hexanal	0.720	0.720	- *	0.500	0.500	−0.500	- *
2-Hexen-1-ol	0.721	0.721	- *	0.500	0.500	−0.500	- *
Benzaldehyde	0.721	0.721	- *	0.500	0.500	−0.500	- *
D-Limonene	0.721	0.721	- *	0.500	0.500	−0.500	- *
Nonanal	0.728	0.728	- *	0.510	0.510	−0.510	- *

* The value of the correlation coefficient was found to be equal to one which is statistically unrealistic, indicative however, of a very strong correlation.

**Table 7:** Correlation between volatile compounds and physicochemical/sensory attributes in the Skeena cultivar.

SKEENA	Flavour	Texture	TA	pH	TSS	TPC	Colour
Acetaldehyde	0.188	0.188	- *	−0.993	−0.501	−0.501	−0.499
Ethanol	0.189	0.189	- *	−0.993	−0.500	−0.500	−0.500
2-Propanone	0.189	0.189	- *	−0.993	−0.500	−0.500	−0.500
2-Methyl-1,3-butadiene	0.187	0.187	- *	−0.993	−0.502	−0.502	−0.498
3-Methyl-3-buten-1-ol	0.188	0.188	- *	−0.993	−0.501	−0.501	−0.499
3-Methylbutan-1-ol	0.192	0.192	- *	−0.994	−0.497	−0.497	−0.503
3 Methyl-2-buten-1-ol	0.190	0.190	- *	−0.994	−0.499	−0.499	−0.501
Hexanal	0.189	0.189	- *	−0.993	−0.500	−0.500	−0.500
2-Hexenal	0.189	0.189	- *	−0.993	−0.500	−0.500	−0.500
2-Hexen-1-ol	0.189	0.189	- *	−0.993	−0.500	−0.500	−0.500
1-Hexanol	0.188	0.188	- *	−0.993	−0.501	−0.501	−0.499
2,4-Hexadienal	0.190	0.190	- *	−0.994	−0.499	−0.499	−0.501
Benzaldehyde	0.189	0.189	- *	−0.993	−0.500	−0.500	−0.500
2-Hexenyl acetate	0.189	0.189	- *	−0.993	−0.500	−0.500	−0.500
Benzyl alcohol	0.189	0.189	- *	−0.993	−0.500	−0.500	−0.500
Nonanal	0.189	0.189	- *	−0.993	−0.500	−0.500	−0.500

* The value of the correlation coefficient was found to be equal to one which is statistically unrealistic, indicative however, of a very strong correlation.

Due to the small number of experimental observations, the above results are only indicative, awaiting further verification with data to be collected during the upcoming harvesting year. 

For the Canada giant cultivar, it is shown in Table 4 that all volatiles determined, except nonanal, showed a strong positive correlation to pH and a moderate positive correlation to TPC. Likewise, there is a moderate negative correlation of volatiles to texture and colour. Moreover, there was a very strong positive correlation of volatiles to TSS and a very strong negative correlation to TA. Finally, nonanal showed a strong positive correlation to pH and TSS, a strong negative correlation to TA and a moderate negative correlation to texture and colour.

For the Ferrovia cultivar (Table 5) all volatiles determined showed a moderate negative correlation to pH, TPC and colour. They also showed a very strong negative correlation to TA/flavour/texture and a very strong positive correlation to TSS.

For the Lapins cultivar (Table 6) all volatiles showed a strong positive correlation to flavour and texture, a moderate positive correlation to pH/TSS and a moderate negative correlation to TPC. They also showed a very strong negative correlation to TA and a very strong positive correlation to colour.

Finally, for the Skeena cultivar (Table 7) all volatiles showed a strong negative correlation to pH, a moderate negative correlation to TSS/TPC/colour and a very strong negative correlation to TA. As a general trend, cherry cultivars with the highest amount of volatiles (Skeena and Ferrovia) gave the highest flavour and TSS scores. Likewise, Malaman *et al.* [11] reported that Brazilian cherry extracts receiving higher flavour scores gave higher volatile peak intensities.

Volatiles identified in the Ferrovia cultivar are negatively correlated to all physico-chemical and sensory properties and positively correlated only to TSS. In the Lapins cultivar volatiles are positively correlated to all parameters except TA and TPC which show a negative correlation to volatiles. Regarding the Skeena cultivar, where correlations exist between volatiles and physico-chemical parameters (TA, pH, TSS, TPC, colour), these were negative. It is also observed that TA shows a very strong negative correlation to volatiles for all four cherry cultivars. TPC shows a negative correlation to volatiles of the Ferrovia, Lapins and Skeena cultivars and a positive correlation to volatiles of the Canada giant cultivar. TSS shows a positive correlation to volatiles of the Canada giant, Lapins and Ferrovia cultivars and a negative correlation to volatiles of the Skeena cultivar. Finally, colour shows a negative correlation to volatiles of the Canada giant, Skeena and Ferrovia cultivars and a strong positive correlation to those of the Lapins cultivar.

### 2.5. Correlation between Physicochemical and Sensory Attributes

Linear correlation of physicochemical parameters of the four cherry cultivars with sensory attributes is presented in Table 8, Table 9, Table 10 and Table 11.

**Table 8:** Correlation between physicochemical and sensory attributes in the Canada giant cultivar.

CANADA GIANT	Flavour	Texture	TA	pH	TSS	TPC	Colour
**Flavour**	1	0.929	0.327	−0.756	−0.327	−0.982	0.929
**Texture**	0.929	1	0.655	−0.945	−0.655	−0.982	- *
**TA**	0.327	0.655	1	−0.866	- *	−0.500	0.655
**pH**	−0.756	−0.945	−0.866	1	0.866	0.866	−0.945
**TSS**	−0.327	−0.655	- *	0.866	1	0.500	−0.655
**TPC**	−0.982	−0.982	−0.500	0.866	0.500	1	−0.982
**Colour**	0.929	- *	0.655	−0.945	−0.655	−0.982	1

* The value of the correlation coefficient was found to be equal to one which is statistically unrealistic, indicative however, of a very strong correlation.

**Table 9:** Correlation between physicochemical and sensory attributes in the Ferrovia cultivar.

FERROVIA	Flavour	Texture	TA	pH	TSS	TPC	Colour
**Flavour**	1	- *	- *	0.500	- *	0.500	0.596
**Texture**	- *	1	- *	0.500	- *	0.500	0.596
**TA**	- *	- *	1	0.500	- *	0.500	0.596
**pH**	0.500	0.500	0.500	1	−0.500	−0.500	0.993
**TSS**	- *	- *	- *	−0.500	1	−0.500	−0.596
**TPC**	0.500	0.500	0.500	−0.500	−0.500	1	−0.397
**Colour**	0.596	0.596	0.596	0.993	−0.596	−0.397	1

* The value of the correlation coefficient was found to be equal to one which is statistically unrealistic, indicative however, of a very strong correlation.

**Table 10:** Correlation between physicochemical and sensory attributes in the Lapins cultivar.

LAPINS	Flavour	Texture	TA	pH	TSS	TPC	Colour
**Flavour**	1	- *	−0.721	0.961	0.961	−0.961	0.721
**Texture**	- *	1	−0.721	0.961	0.961	−0.961	0.721
**TA**	−0.721	−0.721	1	−0.500	−0.500	0.500	- *
**pH**	0.961	0.961	−0.500	1	- *	- *	0.500
**TSS**	0.961	0.961	−0.500	- *	1	- *	0.500
**TPC**	−0.961	−0.961	0.500	- *	- *	1	−0.500
**Colour**	0.721	0.721	- *	0.500	0.500	−0.500	1

* The value of the correlation coefficient was found to be equal to one which is statistically unrealistic, indicative however, of a very strong correlation.

**Table 11:** Correlation between physicochemical and sensory attributes in the Skeena cultivar.

SKEENA	Flavour	Texture	TA	pH	TSS	TPC	Colour
**Flavour**	1	- *	−0.189	−0.300	0.756	0.756	−0.945
**Texture**	- *	1	−0.189	−0.300	0.756	0.756	−0.945
**TA**	−0.189	−0.189	1	0.993	0.500	0.500	0.500
**pH**	−0.300	−0.300	0.993	1	0.397	0.397	0.596
**TSS**	0.756	0.756	0.500	0.397	1	- *	−0.500
**TPC**	0.756	0.756	0.500	0.397	- *	1	−0.500
**Colour**	−0.945	−0.945	0.500	0.596	−0.500	−0.500	1

* The value of the correlation coefficient was found to be equal to one which is statistically unrealistic, indicative however, of a very strong correlation.

As mentioned previously, due to the small number of experimental observations, the results are only indicative, awaiting further verification with data to be collected during the upcoming harvesting year. 

For the Canada giant cultivar, it is seen in Table 8 that flavour showed a strong positive correlation to texture and colour and a strong negative correlation to pH and TPC. Texture showed a moderate positive correlation to TA, a strong negative correlation to pH, TPC and a moderate negative correlation to TSS. TA showed a strong negative correlation to pH and a moderate negative and positive correlation to TPC and colour respectively. In turn, pH showed a strong positive correlation to TSS and TPC and a strong negative correlation to colour. TPC showed a strong negative correlation to colour. Furthermore, there was a very strong negative correlation of TA to TSS and a very strong positive correlation of texture to colour. 

For the Ferrovia cultivar, it is seen in Table 9 that pH showed a strong positive correlation to colour. Also, flavour showed a very strong positive correlation to texture and TA and a very strong negative correlation to TSS. Texture showed a very strong positive correlation to TA and a very strong negative correlation to TSS. Finally, TA showed a very strong negative correlation to TSS. 

For the Lapins cultivar, it is seen in Table 10 that flavour showed a strong positive correlation to pH/TSS/colour and a strong negative correlation to TA and TPC. Finally, texture showed a strong positive correlation to pH/TSS/colour and a strong negative correlation to TA/TPC. Flavour showed a very strong positive correlation to texture, while TA showed a very strong negative correlation to colour. pH showed a very strong positive correlation to TSS and a very strong negative correlation to TPC. Finally, TSS showed a very strong negative correlation to TPC.

For the Skeena cultivar, it is seen in Table 11 that flavour and texture showed a strong positive correlation to TSS/TPC and a strong negative correlation to colour. TA showed a strong positive correlation to pH. Finally, TSS showed a very strong positive correlation to TPC, while flavour showed a very strong positive correlation to texture.

It has been reported that TSS and TA are much related to intensity of cherry flavour and that consumer acceptability increases with high TSS and TA levels [2,8]. According to Chauvin *et al.* [35] overall acceptance of “Sweetheart” cherry cultivar was strongly correlated to flavour intensity. In another study [2], it was reported that consumer acceptance of “Brooks” and “Bing” cherries was highly dependent on TSS. In order to satisfy the majority of American consumers TSS needed to be at least 16%. Kappel *et al.* [37] found a linear relationship between TSS and cherry flavour suggesting a minimum content of 15% for optimal acceptance of sweet cherries.

## 3. Experimental Section 

### 3.1. Cherry Sample Collection

The samples of sweet cherries used in this study were obtained from ten year old sweet cherry trees of four different cultivars (Canada giant, Ferrovia, Lapins and Skeena) from commercial orchards of the Agricultural Cooperative of Naoussa, Greece in late May-early June 2014. The cherry orchards were located 450–500 m above sea level (lat. 40°37'46''N, long. 22°4'5''E). Edible ripe fruits were collected at random from multiple trees. The total weight of cherries collected was 20 kg (5 kg of each of four cultivars). The harvesting criteria for each cherry cultivar were the ground skin colour of the fruit, the region microclimate and the time period between fruit set and ripening. Samples were transported to the laboratory in plastic crates placed in isothermal boxes within 4 h of harvesting and stored at 4 °C until analysis.

### 3.2. Physicochemical Analysis

Three independent samples of twenty fruits, each from the four cultivars, were homogenized after removing the pit in a Moulinex home blender. TSS were measured at 20 °C with a model ATC portable refractometer (Atago, Tokyo, Japan) and the results were expressed as °Brix. The pH was measured using a Delta OHM, model HD 3456.2, pH-meter (Padova, Italy). Ten grams of homogenized sample were centrifuged at 8000 rpm for 10 min at 4 °C (Biofuge Primo R, Heraeus, Kendro Laboratory Products GmbH, Hanau, Germany). The supernatant was used for the determination of titratable acidity (TA). Five mL of the supernatant was diluted with distilled water at 1:10 ratio and this solution was used for the determination of titratable acidity with 0.1 N NaOH. TA was expressed as malic acid equivalents per 100 g FW.

### 3.3. Total Phenolic Content (TPC)

Five grams of smashed cherries and 30 mL of acidic methanol-water solution (80:20, v/v pH 3) were placed in a centrifuge tube. The tube was vortexed (vortex ZX3 Velp Scientifica, Milano, Italy) for 1 min and centrifuged at 8000 rpm for 15 min at 4 °C. Another 20 mL of acidic methanol-water solution (80:20, v/v pH 3) were added to the residue, followed by stirring and centrifugation. The supernatants of the two centrifugations were combined. From the determination of TPC 0.2 mL of the methanolic extract were mixed with 2.3 mL distilled water and 0.25 mL Folin–Ciocalteu reagent (Merck, Darmstadt, Germany). After 3 min 0.5 mL of 20% w/v sodium carbonate solution were added. Water was added to reach a final volume of 5 mL. The mixture was stirred and was kept for 2 h at room temperature in the dark. The absorbance was measured at 725 nm with a Lambda 25 UV/VIS Spectrometer, Perkin Elmer (Waltham, MA, USA). Aqueous solutions of gallic acid (concentrations between 70 and 250 ppm) were used for quantification of TPC. Results were expressed as mg of gallic acid equivalents per 100 g of fresh weight (mg GAE/100 g FW).

### 3.4. Determination of Volatiles

#### 3.4.1. Solid Phase Microextraction (SPME) Sampling

Experimental parameters including fiber type (DVB/CAR/PDMS 50/30 μm, CAR/PDMS 75 μm, PDMS 100 μm, PDMS/DVB 65 μm), sample weight (1 and 2 g), headspace volume of the vial (10 mL and 20 mL), equilibrium time (5, 10 and 15 min), sample temperature (40 and 45 °C) and fiber exposure time (10, 15, 20 and 30 min) were optimized. The optimized parameters are given in the following procedure:

Solid cherry samples of 1 g, 0.2 g NaCl, 10 µL of internal standard 4-methyl-2-pentanone (8 µg/L) (Sigma–Aldrich Co., Munich, Germany) and a microstirring bar were placed in a 10 mL glass vial and sealed with an aluminum crimp-cap equipped with a Teflon-coated needle-pierceable septum supplied by Supelco Co. (St. Louis, MO, USA). Solid-phase microextraction (SPME) was performed with a 75 µm CAR/PDMS fiber (Supelco). The vial was placed in a water bath thermostated at 45 °C and stirred at 8000 rpm. After allowing 5 min for the sample to equilibrate, the needle of the SPME device was inserted into the vial through the septum and the fiber was exposed to the headspace of the sample. After 20 min of exposure, the fiber was transferred to the injection port of a gas chromatograph.

#### 3.4.2. Gas Chromatography–Mass Spectrometry Analysis

An Agilent 7890A series gas chromatograph equipped with an Agilent 5975C mass selective detector (USA) was used for the analysis of volatile compounds adsorbed onto the SPME fiber. The column used was a DB-5 MS (60 m × 0.320 mm i.d. and 1 µm film thickness, J & W Scientific, Agilent Technologies, Santa Clara, CA, USA). The flow rate of the helium carrier gas was 1.5 mL/min. The injector temperature was 260 °C in split mode (2:1). The SPME fiber remained in the injector for 10 min. The initial temperature of column was 40 °C, held for 2 min, heated to 140 °C at a rate of 5 °C/min, heated to 250 °C at a rate of 10 °C/min and held at 250 °C for 2 min. MS conditions were as follows: Source temperature: 230 °C; Quadrupole temperature: 150 °C; transfer line temperature: 270 °C; acquisition mode electron impact (EI 70 eV) and mass range *m/z*: 30–350. Identification of volatile compounds was achieved by comparison of mass spectra of eluting compounds to those of the Wiley library [38]. In addition, the retention index (RI) values of volatile compounds were calculated using n-alkane (C_8_–C_20_) standard (Fluka, Buchs, Switzerland), as well as C_5_‒C_7_ alkanes dissolved in hexane (Fluka). Semi-quantification of volatiles was achieved by comparing the MS detector response of the internal standard to that of the recorded peaks.

### 3.5. Sensory Evaluation

The samples were evaluated, by a 51-member panel (acceptability test). Panelists (28 females, 23 males) were chosen among graduate students and faculty of the Department of Chemistry, University of Ioannina using the following criteria: ages between 18 and 60, non-smokers, who consume cherries regularly during fresh cherry season. Each panelist was served a set of four different samples corresponding to the four cherry cultivars. Attributes evaluated were: skin colour/appearance, texture (firmness), odour and taste (sweetness/sourness) of cherries. The panelists evaluated samples in individual booths under controlled RH (60%), T = 22 °C and natural lighting conditions. They were instructed to cleanse their palate consuming a cracker and sipping water between evaluation of each sample. Acceptability of colour, texture, odour and taste was evaluated using a 5-point hedonic scale with 5 corresponding to the most liked sample and 0 corresponding to the least liked sample. A score of 3 was taken as the lower limit of acceptability [39].

### 3.6. Statistical Methods

The statistical processing of data was performed using the SPSS 23.0 Statistics software [40]. Three different samples from each cultivar were analyzed (n = 3). In order to test the differences between the cherries’ cultivars, with respect to physicochemical and sensory characteristics, One Way Analysis of Variance (ANOVA) was conducted, for each one of the eight dependent variables, used in the analysis (TA, MI, pH, TSS, TPC, Flavour, Texture, Colour). The independent variable is the cherry cultivar, with 4 levels (treatments) (Canada giant, Ferrovia, Lappins and Skeena). This variable is a fixed one, since the four levels have been specifically chosen by the analyst [41]. The same analysis (One Way ANOVA) was conducted, in order to detect differences among cherry cultivars with respect to volatile compounds. The dependent variables were the volatile compounds detected in at least one cultivar.

The results of ANOVAs showed, that almost all the dependent variables are significant for the determination of differences among the cherry cultivars (*p*-values < 0.05). Only flavour was non-significant (*p*-value > 0.05) for this purpose.

In order to specify the differences between the means, we applied multiple comparison methods. The Duncan’s test was used, since all the treatments have the same number of experimental observations. 

The adjusted model has the form:

*Y_ij_ =* µ *+ τ_i_ + ε_ij_*, *i* = 1,2,3,4 *j* = 1,2,3

where µ is a parameter common to all treatments, and τ*_i_* is a parameter unique to the *i*-th treatment, which is actually the effect of the *i*-th treatment (cherry cultivar). *Y_ij_* is the *ij*-th observation *i.e.*, the *j* observation of the *i*-th treatment (volatile compound or physicochemical attribute). ε*_ij_* is the random error of the *ij*-th observation.

Before analysis, the homogeneity of variances was tested by the Levene’s test, where all the ANOVAs seem to satisfy this requirement (*p*-values > 0.05).

To determine correlation coefficients, Pearson correlation coefficient was used. This coefficient is suitable for quantitative variables and varies between −1 and 1. Values at the ends of the interval indicate perfect linear correlation. More specifically, the value −1 indicates perfect linear negative correlation, whereas value 1 indicates perfect linear positive correlation. Values above 0.7 in absolute values, indicate strong relationship, values in the interval 0.4–0.7 in absolute values, show medium linear correlation and <0.4 show weak linear relationship. 

## 4. Conclusions

The present study showed significant differences in physicochemical parameters, sensory attributes and volatile compound composition for all four sweet cherry cultivars grown in Greece. Major sweet cherry volatiles included: 2-hexenal, 2-hexen-1-ol, ethanol, 2-propanone, acetaldehyde, hexanal, benzaldehyde and benzyl alcohol. The contribution of each volatile varied within cultivars. As a general trend, the Ferrovia and the Skeena cultivars gave a higher amount of volatiles indicative of a positive correlation of volatile compounds to higher flavour scores and higher TSS content as compared to the Lapins and the Canada giant cultivars. Furthermore, Skeena cultivar had the highest TPC, among the highest TSS and the lowest TA of all cultivars. In ongoing work, quantitative analysis of volatiles in combination with a substantially higher number of cherry samples will aid to further characterize the differences among the cherry cultivars studied.
